# PET imaging of GABA_A_ receptors in pancreatic islets by [^11^C]flumazenil

**DOI:** 10.1186/s13550-024-01176-5

**Published:** 2024-12-02

**Authors:** Faïza Maloum-Rami, Pierre Cheung, Gunnar Antoni, Zhe Jin, Olof Eriksson, Daniel Espes

**Affiliations:** 1 Department of Medical Cell Biology, Department of Medical Sciences, Science for Life Laboratory, Uppsala University, Box 571, 75123 Uppsala, Sweden; 2 Department of Medicinal Chemistry, Science for Life Laboratory, Uppsala University, Dag Hammarskjölds Väg 14C, 3Tr, 75183 Uppsala, Sweden; 3 PET Center, Center for Medical Imaging, Uppsala University Hospital, Uppsala, Sweden; 4 Science for Life Laboratory, Uppsala University, Uppsala, Sweden; 5 Department of Medical Sciences, Uppsala University, Uppsala, Sweden

**Keywords:** GABA_A_ receptors, PET imaging, Endocrine-cell mass imaging, Flumazenil, Subunits, Type 1 diabetes, Type 2 diabetes, Pancreatic islet, Exocrine pancreas

## Abstract

**Background:**

Type 1 diabetes (T1D) is an autoimmune disease characterized by a progressive β-cell destruction. There are no clinically established methods for quantifying endocrine cells of the pancreas and current knowledge is almost exclusively based on autopsy material and functional measurements. Based on the expression of the γ-aminobutyric acid A receptors (GABA_A_Rs) in pancreatic islets and the fact that GABA_A_R agonists are being explored as treatment for T1D, we hypothesized that the positron emission tomography (PET) tracer [^11^C]flumazenil ([^11^C]FMZ) could serve as a marker of the endocrine mass of the pancreas. The in vivo uptake of [^11^C]FMZ in pig pancreas was evaluated by PET/CT, either tracer alone or after blockade of GABA_A_R by unlabeled flumazenil. The pancreatic binding of [^11^C]FMZ was investigated in vitro with frozen sections of pig pancreas as well as human organ donors, in addition to isolated mouse and human islets and exocrine preparations. The expression of GABA_A_R subunits in pig, human and mouse pancreas was explored by immunohistochemistry.

**Results:**

Strong specific in vivo uptake of [^11^C]FMZ was observed in the pig brain as expected, but in the pancreas the signal was moderate and only partially reduced by blockade. In vitro experiments revealed a positive but weak and variable binding of [^11^C]FMZ in islets compared to exocrine tissue in the mouse, pig and human pancreas. In pig and mouse pancreatic islets we identified the GABA_A_R subunits β2 and γ2 but not α2. In the human pancreas from non-diabetic donors, we have identified the α2, β2 (although weak) and γ2 subunits, whereas from a T2D donor the α2 subunit was missing.

**Conclusions:**

Our findings suggest that [^11^C]FMZ bind to GABA_A_Rs in the islets, but not with a sufficient contrast or magnitude to be implemented as an in vivo PET marker for the endocrine mass of the pancreas. However, GABA_A_Rs with different subunits are widely expressed in the endocrine cells within the pancreas in pig, human and mouse. Hence, the GABA_A_R could still be a potential imaging target for the endocrine cells of the pancreas but would require tracers with higher affinity and selectivity for individual GABA_A_R subunits.

**Supplementary Information:**

The online version contains supplementary material available at 10.1186/s13550-024-01176-5.

## Background

γ-Aminobutyric acid (GABA) is the major inhibitory neurotransmitter in the central nervous system (CNS). The biological functions of GABA are mediated by the activation of GABA receptors. Outside of the CNS, GABA is found in the highest concentrations in human pancreatic β-cells and immune cells, including CD4+ and CD8+ T-cells [1–4]. There are two main types of GABA receptors: GABA_A_Rs which include GABA_A-ρ_ Rs, previously defined as GABA_C_Rs [5, 6] and GABA_B_Rs [7]. GABA_A_Rs are mainly ionotropic receptors [8], while GABA_B_Rs are metabotropic receptors [7]. GABA_A_Rs are hetero-pentamers composed of a variety of subunits with diverse subtypes (α1–6, β1–3, γ1–3, δ, ε_2_, θ, π, ρ1–3) [8] generating a multitude of combinations of GABA_A_Rs translated into activation of different pathways and diverse physiological responses and functions [9, 10]. GABA_A_Rs are generally composed of two α, two β and one γ subunit, with two GABA agonist binding sites, located at the two extracellular interfaces between the adjacent α and β subunits [11]. An additional binding site at the extracellular interface between the α and γ subunits is targeted by benzodiazepines [11, 12], including flumazenil (Suppl. Fig. S1). In the central nervous system (CNS), GABA_A_Rs interact with positive allosteric modulators and negative allosteric modulators, such as flumazenil that has clinical implications by serving as an ‘antidote’ to benzodiazepines since it can reverse their effects. The benzodiazepine competitive antagonist flumazenil radiolabeled with carbon-11 has also been developed as a positron emission tomography (PET) tracer for investigating the regional distribution and occupancy of GABA_A_Rs in the brain. The resulting [^11^C]FMZ is a clinically approved PET-tracer and has been widely and successfully used for studying neurological and psychiatric disorders such as epilepsy [13, 14], dementia [15], Alzheimer’s disease [16] and depressive disorders [17]. In human pancreas, GABA is principally localized to and released by the insulin producing β-cells [18, 19], where it is co-secreted with insulin in a glucose dependent manner by exocytosis from large dense-core vesicles [18]. Once released by the pancreatic β cells, GABA influences hormonal secretion via autocrine and paracrine signaling on endocrine cells and immune cells by activating GABA receptors [1, 18]. GABA_A_ receptors have been found to be present in all endocrine cells of human pancreatic islets [18, 20, 21] (Suppl. Fig. S1) and in several type of immune cells [1, 22, 23]. Gene transcripts encoding the GABA_A_ receptor subunits have been examined in the brain, endocrine pancreas and immune system from different species, revealing a diversity in the combinations of subunit isoforms expressed in each organ and cell types. In addition, inter-species variability of GABA_A_R subunits was found [3, 23].

The aim of the current study was to evaluate whether the radioligand [^11^C]FMZ could be used to noninvasively image GABA_A_Rs in the pancreas, as a potential surrogate marker of the endocrine mass.

## Results

### In vivo imaging of GABA_A_Rs bio-distribution in pig with [^11^C]flumazenil

**Fig. 1:**
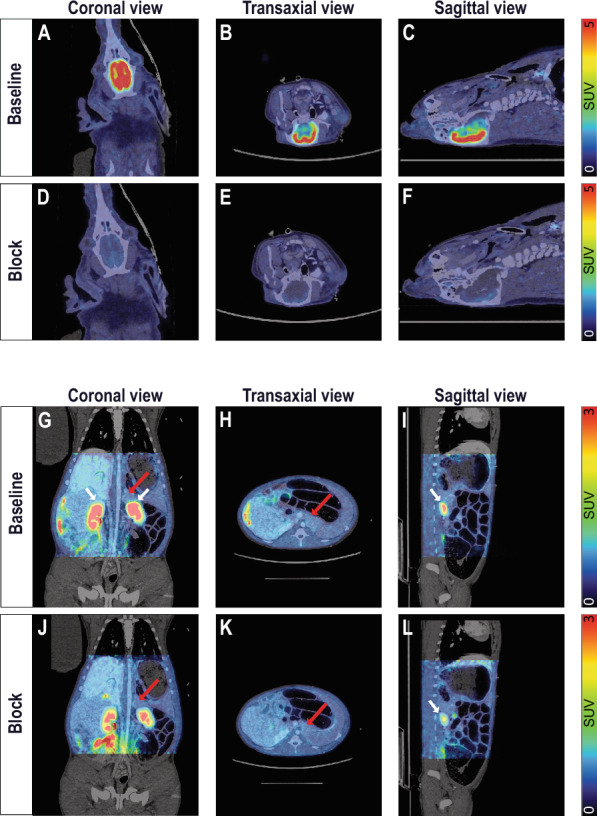
In vivo PET/CT imaging of [^11^C]FMZ for GABA_A_Rs distribution in pig brain and pancreas. Coronal, transaxial and sagittal views of fused PET/CT scans from pig brain (SUV = 5) (**A**–**F**) and pancreas (SUV = 3) (**G**–**L**) showing the averaged signal from 60 to 90 min in the brain and 50–60 min in the pancreas at baseline (**A**–**C** and **G**–**I** respectively) following [^11^C]FMZ administration and after blocking with cold flumazenil (**D**–**F** and **J**–**L** respectively). Red arrows represent pancreas and white arrows represent kidneys

**Fig. 2:**
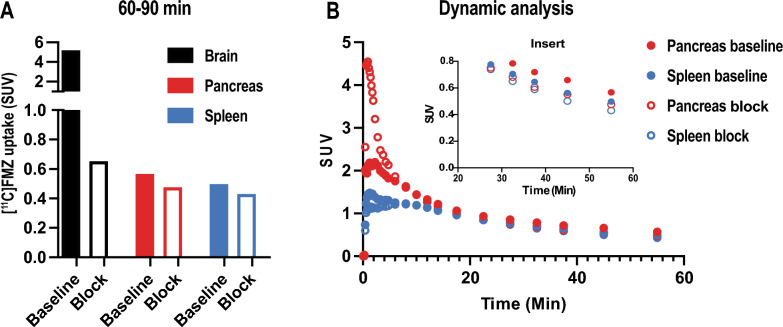
Quantitative analyses of [^11^C]FMZ PET imaging in pig brain, pancreas and spleen. (**A**) [^11^C]FMZ uptake in pig brain, pancreas and spleen at baseline and after blocking with cold FMZ. The binding of the tracer was calculated by averaging the signal from 60 to 90 min. (**B**) Time-activity curves in pig pancreas (red circles) and spleen (blue circles) following [^11^C]FMZ administration at baseline (closed circles) and after blocking with cold FMZ (open circles). The insert shows a focused axis range adapted to the pancreas

An in vivo study targeting GABA_A_Rs for pancreatic endocrine-cell imaging was performed by PET using the GABA_A_R radioligand [^11^C]FMZ in a healthy control pig model (Yorkshire x Swedish Landrace x Hampshire) (n = 1). Pancreas, brain (positive control) and spleen (negative control) uptake of [^11^C]FMZ was evaluated. We observed a strong binding of [^11^C]FMZ in the brain after tracer injection, which was expected, given the high density of GABA_A_R (Fig. 1 A-C and Suppl. Fig. S2 A-C). Spleen and other peripheral GABA_A_R negative tissues correspondingly exhibited negligible uptake of [^11^C]FMZ. The brain uptake was nearly completely abolished by pre-injection of cold flumazenil, demonstrating almost exclusively receptor mediated binding (Fig. 1 D-F and Suppl. Fig. S2 D-F). In the pancreas, however, only moderate binding of [^11^C]FMZ was observed, which was slightly above the observed background in spleen (Fig. 1 G-I,red arrows and Suppl. Fig. S2 B). In the blocking experiment, the [^11^C]FMZ binding signal in the pancreas was reduced only by roughly 20%, indicating that the majority of binding was non-specific (Fig. 1 J-L, red arrows and Fig. 2 A-B).

### In vitro [^11^C]FMZ binding in pig, human, and mouse pancreatic tissue.

**Fig. 3:**
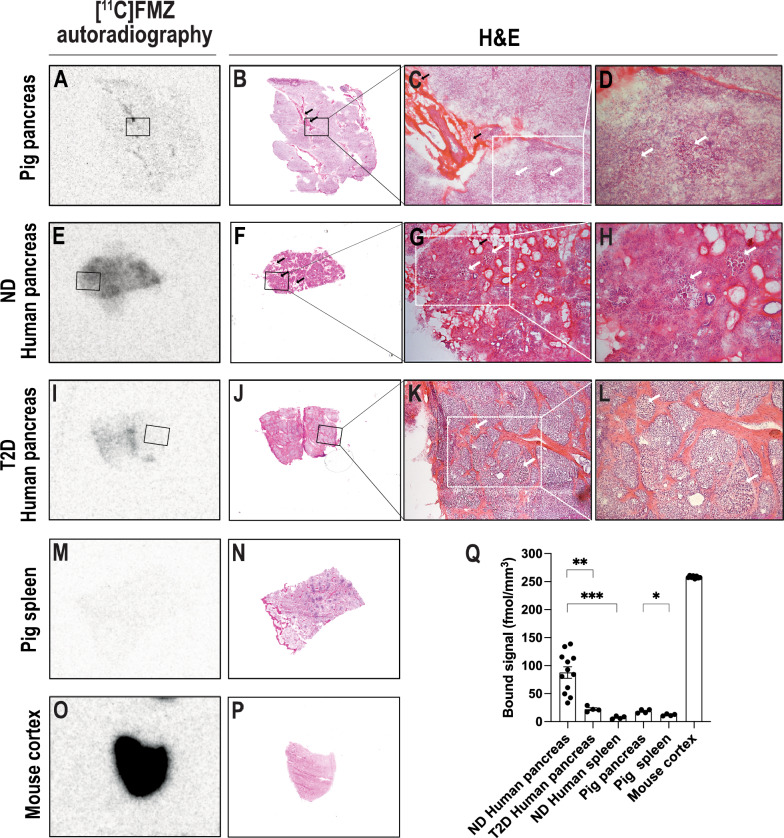
In vitro autoradiography assays of [^11^C]FMZ. Representative images of in vitro autoradiography of [^11^C]FMZ and H&E staining performed on frozen pancreatic sections from control pig (n = 1) (**A**–**D**), human ND donors (n = 3) (**E**–**H**) and T2D donor (n = 1) (**I**–**L**). Moderate hotspots scattered throughout the tissue could be detected in the pig pancreas (**A**). There was at least in part an overlap with the [^11^C]FMZ hotspots and islets (**C** and **D**, white arrows) but there were also areas corresponding to exocrine tissue and adipocytes (**B** and **C**, black arrows). The binding of [^11^C]FMZ in these hotspots was significantly higher compared to the apparent background signal of the negative control from pig spleen (**M** and **Q**). Binding of [^11^C]FMZ was observed in pancreatic sections from non-diabetic human organ donors (**E**). As for the pig pancreas the binding was however not exclusive to pancreatic islets (**F**–**H**). In the T2D donor pancreas an overall lower radiotracer binding intensity was observed compared to pancreas from non-diabetic donors (**I**). No correlation of [^11^C]FMZ uptake and pancreatic islets could be observed in pancreatic sections from the T2D donor (**J**–**L**). Tissue sections from pig spleen (**M**–**N**) and mouse cortex (**O**–**P**) were used as negative- and positive controls of the autoradiography respectively. Scale bar 200 µm (**C**, **G**, **K**) and 400 µm (**D**, **H**, **L**) for magnification of H&E staining. Quantitative analyses of [^11^C]FMZ binding signal to human, pig and mouse tissue sections (**Q**)

The strong presence of GABA_A_Rs in pancreatic islets has been previously established, but surprisingly we observed only a moderate binding of [^11^C]FMZ in the pancreas in vivo. Therefore, we proceeded to investigate the in vitro binding of [^11^C]FMZ to GABA_A_Rs by autoradiography of tissue sections from pancreas (n = 1 healthy pig pancreas, n = 3 non-diabetic and n = 1 T2D human organ donors) (Suppl. Table S1). Mouse brain cortex (n = 1) was used as a positive control due to its high expression of GABA_A_Rs. Splenic tissue from one non-diabetic human organ donor and one healthy pig was used as negative control. The [^11^C]FMZ binding to pig pancreas sections displayed a number moderate hotspots scattered throughout the tissue (Fig. 3A). The binding of [^11^C]FMZ in these hotspots was significantly higher compared to the apparent background signal of the negative control from pig spleen (18.41 ± 1.69 vs.11.94 ± 0.87 fmol/mm^3^, p < 0.05) (Fig. 3M, Q). The tissue sections were stained with H&E in order to locate the pancreatic islets. We found that there was at least in part an overlap with the [^11^C]FMZ hotspots and islets (Fig. 3C, D, white arrows) but there were also areas corresponding to exocrine tissue and adipocytes (Fig. 3B, C, black arrows). In human pancreas from non-diabetic donors, we observed a significantly stronger binding of [^11^C]FMZ when compared with pancreatic sections from the T2D donor (87.61 ± 10.21 vs. 22.25 ± 2.38 fmol/mm^3^, *p* < 0.01) (Fig. 3E, I, Q). As expected, uptake in human spleen sections was lower than in human non-diabetic pancreas (7.20 ± 1.27 *vs.* 87.61 ± 10.21 fmol/mm^3^, *p* < 0.001) (Fig. 3Q).

**Fig. 4:**
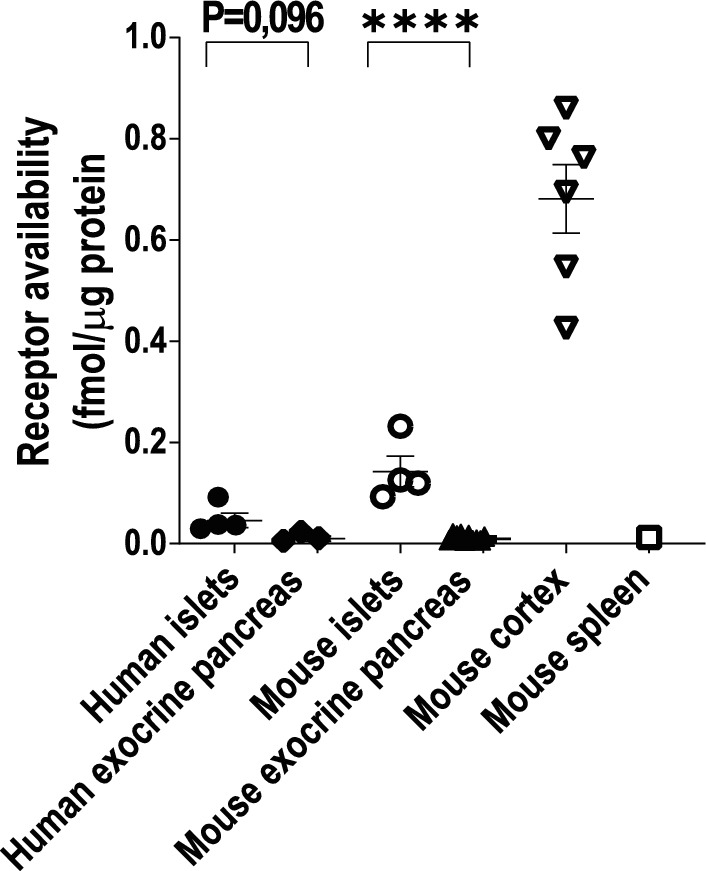
In vitro homogenates binding assay of [^11^C]FMZ. Homogenates of purified islets (n = 4) and exocrine pancreatic cell fractions (n = 3) from non-diabetic human organ donors and isolated islets (n = 4), exocrine pancreatic cell fractions (n = 10), cortex (n = 3) and spleen (n = 1) from control mice were incubated with 0.25 MBq [^11^C]FMZ. All individual experiments were analyzed in duplicate or triplicates. Differences in receptor availability quantification were analyzed using impaired *t* test: *p < 0.05, **p < 0.01, ***p < 0.001. All values are presented as mean ± SEM

To further investigate the specificity of [^11^C]FMZ binding to GABA_A_Rs in the pancreas, we performed radioligand binding assays in homogenates of purified human pancreatic islets (n = 4) and exocrine cell fractions (n = 3) as well as from isolated mouse islets (n = 4) and exocrine cell fractions (n = 6). Mouse brain cortex (n = 3) and spleen (n = 1) were used as positive and negative controls respectively. Binding was determined in all samples after incubation with 2.5 MBq/mL [^11^C]FMZ (50 nM). As expected, the [^11^C]FMZ binding in mouse brain cortex was significantly higher compared with mouse and human pancreatic tissue (Fig. 4). Although far lower than in brain cortex, the [^11^C]FMZ binding was significantly higher in isolated mouse islets compared to exocrine tissue (0.14 ± 0.03 vs. 0.009 ± 0.002 fmol/µg, < 0.001). In human islets, the binding of [^11^C]FMZ displayed a tendency for higher binding when compared with human exocrine tissue (0.046 ± 0.014 vs. 0.01 ± 0.005 fmol/µg, *p* = 0.097) (Fig. 4).

### Immunohistochemistry of GABA_A_R subunits in the pancreas

**Fig. 5:**
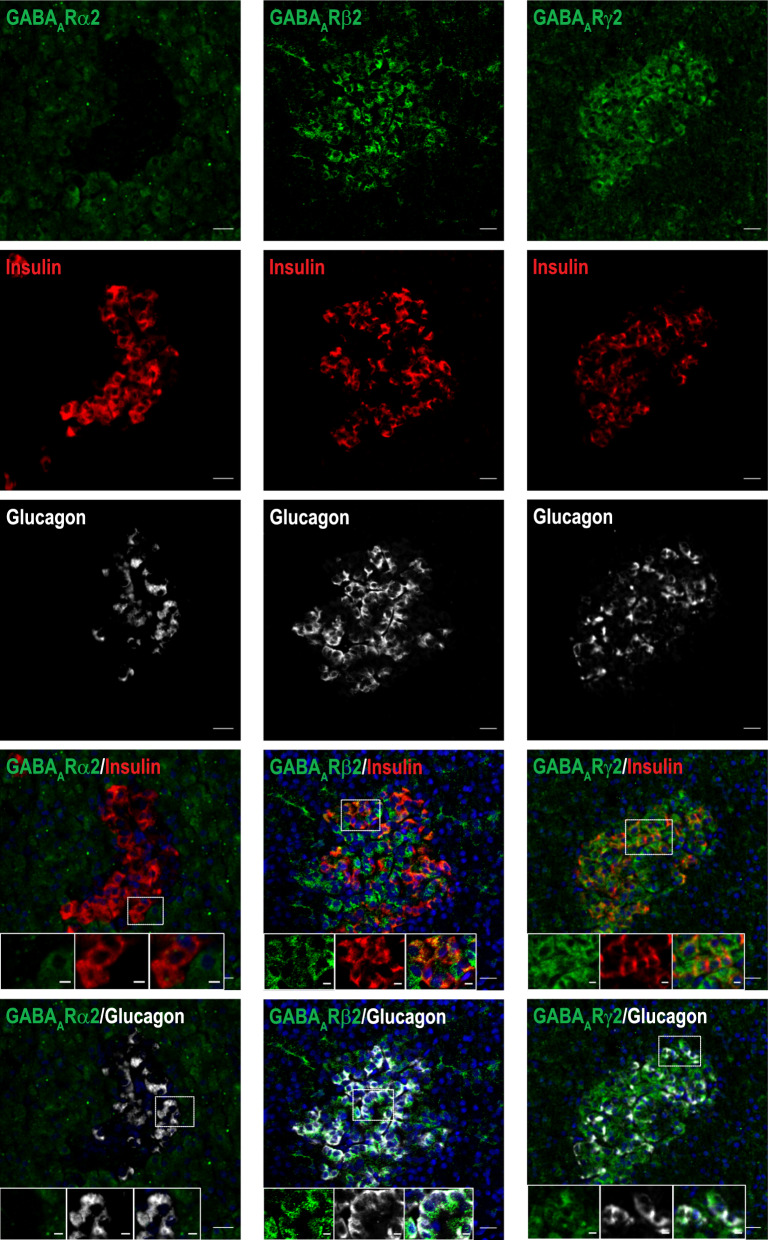
Expression profile of GABA_A_R α2, β2, γ2 subunits in pig pancreatic islet β- and α-cells. Pig pancreatic tissue (n = 4) was co-stained with antibodies for GABA_A_R subunits α2 (left panel), β2 (middle panel), γ2 (right panel) (green), insulin (red), glucagon (white), nuclei (blue). Scale bar 20 µm and 5 µm for magnification

**Fig. 6:**
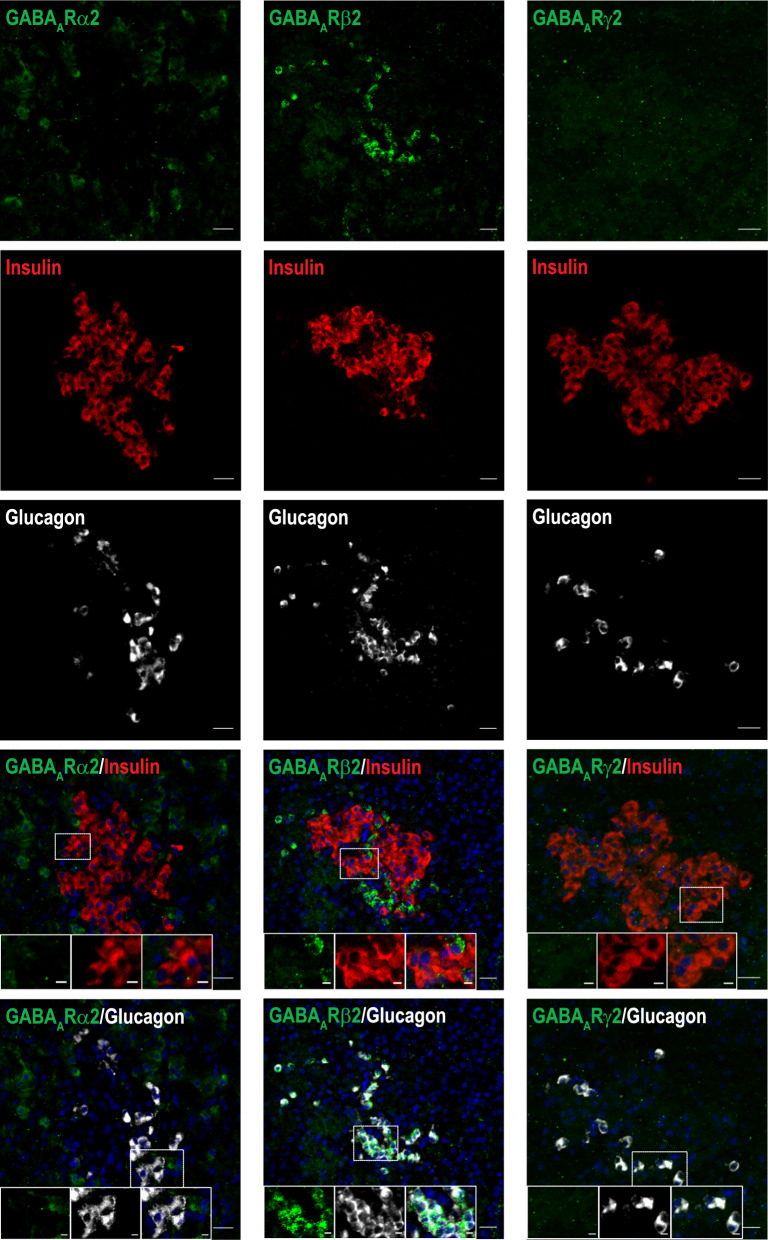
Expression profile of GABA_A_R α2, β2, γ2 subunits in pancreatic islet β- and α-cells of [^11^C]FMZ-injected pig. Pancreatic tissue (n = 1) was co-stained with antibodies for GABA_A_R subunits α2 (left panel), β2 (middle panel), γ2 (right panel) (green), insulin (red), glucagon (white), nuclei (blue). Scale bar 20 µm and 5 µm for magnification

As flumazenil is not selectively targeting a specific GABA_A_R subunit, we used immunohistochemistry to explore which of the GABA_A_R subunits are present in the endocrine cells of pancreatic islets (i.e. α-, β-, and δ-cells) in different species. In pancreas sections from healthy control pigs (n = 4) and the [^11^C]FMZ-injected pig (n = 1), we investigated the presence of α2, β2 and γ2 GABA_A_R subunits, identifying both the β2 and γ2 subunits in β- and α-cells (Fig. 5 middle and right panels respectively), but not in δ-cells (Suppl. Fig. S3, middle and bottom panels respectively, and Suppl. Table S3). In the [^11^C]FMZ-injected pig, we observed no positive staining of the α2 or γ2 subunits in pancreatic islets (Fig. 6) and additionally found in sections from the control pigs that the endocrine islet cells do not stain positively for the α2 subunit (Fig. 5, left panel and Suppl. Fig. S3, top panel). While this suggests lack of α2 presence, it also indicates that the binding sites of flumazenil overlap with the γ2 subunits in the pancreas, as the high dose of cold flumazenil is blocking receptor accessibility during the PET imaging study. This aligns with the previously described binding site for flumazenil and benzodiazepines also in other tissues [11, 12]. In line with that, the β2 subunit could still be detected in islets but was primarily identified in α-cells (Fig. 6).

In human pancreas from non-diabetic organ donors, the GABA_A_R α2 subunit was identified in islets with a heterogeneous staining pattern (Suppl. Fig. S4, left panel). The α2 subunit was primarily detected in β-cells (Suppl. Fig. S4, left panel and Suppl. Fig. S5 A, top panel). The β2 subunit was only weakly detected in islets (Suppl. Fig. S4, middle panel) and preferentially identified in α-cells (Suppl. Fig. S4, middle panel) and δ-cells (Suppl. Fig. S5 A, middle panel). However, some exocrine cells surrounding the islets were also positives for the β2 subunit. The γ2 subunit was identified in islets and could be detected in both β- and α-cells (Suppl. Fig. S5, right panel) as well as δ-cells (Suppl. Fig. S5 A, bottom panel). The γ2 subunit was, however, also detected in the exocrine pancreas (Suppl. Fig. S4, right panel).

Given the reduced [^11^C]FMZ binding observed with autoradiography in pancreatic sections from a T2D donor, we further explored by immunohistochemistry the expression and localization of GABA_A_R α2, β2 and γ2 subunits in pancreatic sections from T2D donors (Suppl. Table S1). However, due to limited access of material the stainings for different subunits were performed using two different donors (n = 1 for α2 and γ2 and n = 1 for β2). The α2 subunit could not be detected in islets from the T2D donor (Suppl. Fig. S6, left panel and Suppl. Fig. S5 B, top panel). The β2 subunit was weakly stained in islets and was preferentially identified in α-cells (Suppl. Fig. S6, middle panel) and δ-cells (Suppl. Fig. S5 B, middle panel). The γ2 subunit was identified in islets from a T2D donor but the staining was overall weak (Suppl. Fig. S6, right panel). We could not identify the γ2 subunit in δ-cells (Suppl. Fig. S5 B, bottom panel).

In mice, we did not find the α2 subunit in islets, neither in β- or α-cells (Suppl. Fig. S7, left panel) nor δ-cells (Suppl. Fig. S8, top panel). The α2 subunit was instead detected in the exocrine pancreas of mice (Suppl. Fig. S7, left panel). However, both the β2 and γ2 subunit was clearly expressed in mouse islets and could be identified in both β- and α-cells (Suppl. Fig. S7) as well as in δ-cells (Suppl. Fig. S8).

## Discussion

In T1D the endocrine mass of the pancreas is affected due to the progressive immune mediated loss of β-cells. In T2D, on the other hand, β-cell function is impaired but in later stages also the mass of β-cells can be reduced [24]. However, most of our knowledge regarding alterations of endocrine cells of the pancreas is based on either autopsy data or functional measures of hormone secretion, which may or may not accurately reflect the anatomical mass of endocrine cells. This is largely explained by the risks associated with acquiring pancreas biopsies and hence, our knowledge of how the endocrine mass is affected during the development and at later stages of diabetes is limited. Currently used markers of metabolic control, i.e. glucose levels, continuous glucose measurements, HbA1c etc., can be used to ensure the fulfilment of treatment criteria, but fail to reflect β-cell mass in both T1D and T2D since these markers are primarily affected by the treatment regime (i.e. exogenous insulin in T1D and insulin and/or oral antidiabetic drugs and GLP-1 analogs in T2D). The establishment of a technique that would enable accurate monitoring and quantification of the endocrine mass of the human pancreas would hence be of major importance in order to increase our understanding of the pathogenesis of diabetes. Therefore, the combination of functional measurements using standardized metabolic tests in concert with assessment of endocrine mass reduction are essential to providing a better understanding the relationship and differential roles between endocrine cell mass and function in diabetes pathogenesis. There is currently a great interest in developing non-invasive imaging methods to enable in vivo quantification of the pancreatic endocrine mass in the early- and late stages of both T1D and T2D [25, 26]. This would be of major importance and especially timely, since in parallel the discovery of biomarkers and autoantibodies associated with T1D have now spurred the discussion of whether or not there should be screening programs for T1D. Linked to this, is an exciting development of novel disease modifying therapies intended to halt the destruction of β-cells in the pre-symptomatic phase of T1D. A challenge with the currently used biomarkers is however that they cannot with certainty identify which individuals will actually progress towards manifest T1D. In this setting, and especially in the early era of screening, imaging could play an important role in identifying those who actually have an ongoing destruction of β-cell mass. Interestingly, immune cells also express GABA receptors but considering the low binding of [^11^C]FMZ in the spleen, which is rich in immune cells, the limited amount of infiltrating immune cells in the pancreas in T1D would most likely not be possible to detect. Due to the presence of GABA_A_Rs in pancreatic islets, we investigated the potential to use the GABA_A_R as an imaging target and surrogate marker for the endocrine cells of the pancreas by adopting the clinically applicable GABA_A_R radioligand [^11^C]FMZ. Experimental in vivo PET scanning in pig revealed, as expected, a strong uptake of [^11^C]FMZ in the pig brain, whereas only a moderate signal in the pancreas. Furthermore, blocking experiment with cold flumazenil resulted in a clear reduction of signal in the brain (≈ 90%), which confirmed the specificity and affinity of [^11^C]FMZ to GABA_A_Rs, but the blocking in the pancreas was limited (≈ 20%).

Due to the low in vivo signal in pancreas, no further animal studies were performed for ethical reasons, and instead we opted to investigate the binding of [^11^C]FMZ by in vitro autoradiography, which revealed hotspots of [^11^C]FMZ binding in the pancreas. These were however not exclusive to the pancreatic islets, which suggests that exocrine and other cells within the pancreas also express GABA_A_Rs [27, 28].

In vitro binding of [^11^C]FMZ in pig pancreas thus revealed a higher concentration of the radioligand on the target tissue in comparison to the binding in pancreas detected by the in vivo PET imaging study. The lower [^11^C]FMZ signal in the pancreas in vivo is likely due to the technical performance of the PET/CT scanner compared to the phosphorimager plates and gamma counter used for the in vitro assays. The spatial resolution (approximately 4 mm) of the clinical PET scanner is not optimal for the relatively low signal from the pig pancreas. Furthermore, the pancreatic islets are far smaller (diameter of ≈150 µm) than the spatial resolution of the scanner, and are additionally distributed heterogeneously across the tissue – likely leading to a diffuse signal prone to be overcome by noise. The PET technology generally has remarkable sensitivity, but the efficiency of gamma counters and autoradiography will still far outperform the PET/CT scanner (Discovery MI, from around 2017) used in this study. The emerging whole body PET scanners are known to have very large increase in sensitivity compared to traditional PET/CT scanners, and thus could be an improved alternative to detect the islet specific signal of [^11^C]FMZ.

Another limitation in the binding of the [^11^C]FMZ might be in part due to the different isoforms and subunits expressed in the brain compared to the pancreas. Furthermore, despite the presence of α2 and γ2 subunits on GAB_A_A receptors, depending on composition of the adjacent subunits on the receptor, the BDZ binding site will be more or less sensitive.

The homogenate binding assay revealed that the islet-to-exocrine ratio is significantly higher in mice. A difference was also observed in human tissue but less pronounced. The differences in binding affinities to purified mouse and human islets compared to their respective exocrine tissue may, at least in part, be explained by the differences inherent with the cell fraction preparation. While isolation of purified mouse islets and exocrine tissue is processed directly after sacrifice, the time of isolation varies from one donor to another in the clinical setting, which can affect the isolation outcome [29]. Whereas mouse pancreatic islets are quite homogenous, and the isolation process is fairly stable, human islets are more heterogenous, both in terms of their cellular composition and function and the isolation process is affected by differences in organ procurement for different donors [30]. Obviously there is also a much higher level of variability between human donors considering the differences in age, gender and BMI etc. which, in addition to the isolation process, will affect the islets quality and physiology. Future studies may consider increasing the number of human samples, which could provide more accurate analyses and representative of the population. In addition, species differences [23] in GABA_A_R subunit expression and their respective binding affinity for [^11^C]FMZ could affect the results. GABA_A_Rs expression and function have previously been documented in rodent pancreatic islets [18, 31] and in islets from non-diabetic and T2D humans [18, 20, 21]. GABA_A_Rs are composed of two α _(1–6)_, two β _(1–3)_ and one of the γ2, π or ρ2 subunits [9, 20], generating multiple possible combinations of GABA_A_R subtypes. Flumazenil binds to the extracellular interfaces between α and γ subunits of the GABA_A_Rs and interacts with GABA_A_Rs composed of α1βγ2, α2βγ2, α3βγ2, α5βγ2, as well as α4βγ2 and α6βγ2 subtypes [9, 32]. From our findings, pancreatic islets in pigs and mice do not express the α2 subunit, whereas we did identify the β2- and γ2 subunit. Hence, islets from both pigs and mice seem to be composed of the α*x*β2γ2 GABA_A_R subtypes, where ‘*x*’ represents one of the α isoforms 1–6 that we did not identify (Fig. 5). In the pancreas of non-diabetic human organ donors we found both the α2 and γ2 subunits, but only to a lower extent the β2 subunit. Depending on which of the α subunit isoforms are expressed, the α*x*β2γ2 GABA_A_R will exhibit distinct physiological and pharmacological properties and can hence affect the binding of [^11^C]FMZ. In addition, we observed (although weak) staining of both the α2 and γ2 subunits in the exocrine pancreas in both pigs and humans (Fig. 5 and Suppl. Fig. S4), suggesting that also the endocrine specificity of [^11^C]FMZ would be limited. The majority of GABA_A_R subunit genes have been detected in the endocrine cells of the human pancreatic islets, with prominent expression of α1, α2, β2, β3, γ2, π and ρ2 [21] out of which the insulin-producing β-cells mainly express α2, β3 and γ2 [18, 20]. Consistent with the aforementioned studies, we found that all the α2, β2 and γ2 subunits were present in islets from non-diabetic human organ donors. While in T2D pancreas we could not detect the α2 subunit in islets and the staining of both β2 and γ2 was reduced. The reduction or absence of different GABA_A_R subunits observed in T2D islet cells is concordant with the reduction in [^11^C]FMZ binding to T2D pancreatic sections, observed by in vitro autoradiography. Due to the restricted number of human samples from healthy donors and T2D patients, our findings provide relevant but still explorative data regarding the specificity of binding of [^11^C]FMZ in vitro in human endocrine pancreas and the expression of islet GABA_A_R subunits. Considering the heterogeneity of pancreatic islets in human [30], the variability between different individuals and especially in the context of T2D [33], future studies with an increased number of observations from human samples would be needed in order to verify the current findings.

In mouse islet cells, α4, β3, δ and γ2 GABA_A_R subunits were predominantly identified [20, 34, 35]. Consistent with these studies we found that none of the endocrine islet cells express the α2 subunit in mice. Inversely β2 and γ2 subunit were expressed in all examined islet endocrine cells (*i.e.* α-, β- and δ-cells).

A strength of the current study is that it includes both in vivo and in vitro experiments and that species differences regarding expression of GABA_A_R subunits was explored. There are however several limitations, the most apparent being the limited number of observations. Also, the immunohistochemistry examinations were explorative and not quantitative and limited especially in the number of examined human samples, which could be especially of importance for findings in T2D considering the great heterogeneity of the disease.

Since flumazenil interacts non selectively with multiple GABA_A_R subtypes, it could be of interest to develop a receptor subtype-selective radioligand, specific for the subunits expressed in the endocrine cells of the pancreas. Based on the gene expression of GABA_A_R subunits in β-cells from both non-diabetic and T2D donors the most interesting subunits would be α2, β3 and γ2 [20].

Taken together, these results suggest that [^11^C]FMZ is unsuitable as a marker for the pancreatic endocrine mass, both due to its low uptake in vivo and to the presence of GABA_A_Rs in the exocrine pancreas. However, by developing new tracers that target specific GABA_A_R subunits that are predominantly expressed by the endocrine pancreas may provide a possibility to target pancreatic islets.

## Conclusions

Our findings suggest that [^11^C]FMZ binds to GABA_A_Rs in pancreatic islets, but not with a sufficient contrast or magnitude to be implemented as an in vivo PET marker for the endocrine mass of the pancreas. However, GABA_A_Rs with different subunits are widely expressed in the endocrine cells within the pancreas in pig, human and mouse. Hence, the GABA_A_R could still be a potential imaging target for the endocrine cells of the pancreas but would require tracers with higher affinity and selectivity for individual GABA_A_R subunits.

## Methods

### Radiochemistry

[^11^C]FMZ was synthesized according to clinical routine at Uppsala University Hospital, as described previously [36]. Different batches of [^11^C]FMZ were delivered with molar activity at end of synthesis of approximately 300 MBq/nmol for PET imaging studies and approximately 150 MBq/nmol for in vitro experiments.

### Animal preparation and handling

For the PET/CT imaging study, one male pig (weight 24.5 kg) (Yorkshire x Swedish Landrace x Hampshire) was used. The pig was raised at a farm and transported to the PET facility at the day of experiment. Upon arrival it was sedated and prepared for the PET/CT experiment as previously described [37]. Briefly, the pig was sedated by intra-auricular infusion of combined ketamine and fentanyl (free from anesthetic benzodiazepine to prevent any interference with [^11^C]FMZ and blocking compound). The pig was connected to central venous catheter for the infusion of [^11^C]FMZ, cold flumazenil and contrast compound. All animal procedures were performed in agreement with the Uppsala University guidelines for animal research (UFV 2007/724) and national legislations. The studies were planned and performed in according to the 3Rs principle and to the ARRIVE guidelines for animal research and were approved by the animal Research Ethical Committee of the Uppsala Region (Ethical approval #5.8.18–15,648/2019).

### In vivo biodistribution study with [^11^C]FMZ in pig model

The imaging procedures were performed using a Discovery MI PET/CT scanner (GE Healthcare), with a 25 cm field of view. A CT acquisition was first performed without contrast as previously described (Cheung et al., 2021) before a bolus intravenous injection of approximately 12 MBq/kg of [^11^C]FMZ dissolved in 25 mL of PBS (pH 7.4). A dynamic PET imaging of 60 min was subsequently acquired using the following parameters: 30 frames of 12 × 10, 6 × 30, 5 × 120, 5 × 300 and 2 × 600 s, VPFX-S, 3 i/16 s, 256 × 256 × 89 pixels, 3 mm post filter, 500 mm diameter zoom. A whole-body 30 min static PET/CT acquisition was performed after the abdominal scan (CT: 100 kV, 80–400 mA, noise index 10, rotation 0.5′, full spiral, slice thickness 3.75 mm, pitch 0.98:1, recon diameter 50 mm; PET: VPFX-S, 3 i/16 s, 256 × 256 pixels, 3 mm post filter, 500 mm diameter zoom). Three hours after the first injection of [^11^C]FMZ, cold flumazenil was intravenously infused at a dose corresponding to 0.1 mg/kg of pig body weight. Prior to starting a second PET/CT scan following the procedure previously described, a second dose of approximately 12 MBq/Kg of [^11^C]FMZ was intravenously injected. At the end of the dynamic PET scan over pancreas, a contrast enhanced CT acquisition was performed following the procedure described previously (Cheung et al., 2021). The PET/CT imaging data for both the baseline scan and with the addition of non-radioactive FMZ were analyzed using the PBAS tool from the PMOD software 4.3 (PMOD Technologies). Manual segmentation of the pancreas and spleen were performed on the co-registered abdominal CT, whereas the brain was delineated on the whole-body CT. The entire organs (pancreas, spleen, brain) were delineated on CT and transferred to the PET images, but care was taken to avoid spill-in of PET signal from surrounding tissues (mainly kidney in this case). In cases where spill-in was suspected, the segmentations were decreased in size to omit confounding signal from other tissues. The same areas of the tissues were segmented on both the baseline and blocking PET/CT scans.

The tissue uptake in kBq/cc was converted to Standardized Uptake Values (SUV) by dividing with the amount of administered radioactivity (in kBq) per body weight (g). SUV thus has the unit g/mL, but is often considered unitless by approximating the density of tissue to 1 g/mL. At the end of the imaging studies the animal was euthanized following intravenous injection of KCl. Organs of interest (pancreas and spleen) were removed, fixed in PFA 4% and embedded in paraffin for further immunostaining experiments.

#### Human islets and pancreas tissue

Isolated human islets and fractions of exocrine tissue were obtained from the Nordic Network for Clinical Islet Transplantation laboratory in Uppsala, Sweden. Fresh frozen human tissue including spleen and pancreas were provided by Uppsala Biobank. All human sample experiments were approved by the Regional Ethics Board of Uppsala, Sweden (now the Swedish Ethical Review Authority). Characteristics of human tissue and cell fractions obtained from cadaveric donors are listed in Supplementary Table S1. Human islets and fractions of exocrine tissue were hand-picked and collected separately into fresh CMRL1066 medium (# 21530027; Gibco) supplemented with 10% fetal bovine serum (# ref; Gibco), 2 mM L-glutamine (# G7513; Sigma) 100 Units/ml Penicillin, 100 µg/ml Streptomycin (# 1074440 Roche Diagnostics, Mannheim, Germany).

#### Mouse islets and pancreas tissue

Mice were sacrificed by cervical dislocation. Pancreata were perfused with 1 mg/ml collagenase P (# 11213865001; Sigma Aldrich) extracted and digested in Hank’s balance salt solution (HBSS) (Sigma Aldrich) buffered with 25 mM HEPES (# 15630-056; Gibco) (pH 7.4), supplemented with 0,25% (w/v) bovine serum albumin (BSA) (# A7030; Sigma Aldrich). Islets and exocrine pancreas were isolated and hand-picked under a stereo microscope in cold-HBSS buffered with HEPES (pH 7.4) supplemented with 0.5% BSA (w/v). Mouse pancreatic fractions were then collected separately into fresh RPMI 1640 (#R-0883; Sigma Aldrich) supplemented with 10% fetal bovine serum (# F7524; Sigma Aldrich), 2 mM L-glutamine (# G7513; Sigma Aldrich), 100 Units/ml Penicillin, 100 µg/ml Streptomycin (# 1074440 Roche Diagnostics, Mannheim, Germany).

### Human and mouse cell fraction isolation for in vitro homogenates binding assay

Prior to in vitro experimentation, isolated human islets were kept in the same medium to recover overnight in an atmosphere of 95% air and 5% CO_2_ at 37 °C. The exocrine tissue was processed directly after isolation to prevent any enzymatic degradation of the tissue. Human and mouse endocrine and exocrine pancreatic cell fractions and mouse tissue (cortex and spleen) were freshly collected into ice- cold 0,32 M sucrose + BSA (1 mg per 100 ml 0.32 M sucrose) (# A7030; Sigma Aldrich) and homogenized by hand using a Dounce glass homogenizer (# P7734-1EA; Sigma Aldrich). Sample protein concentration was quantified by Bio-Rad Protein Assay (# 500–0006; Bio-Rad Laboratories) using BSA as a standard. Homogenates from human and mouse samples were snap frozen until used for in vitro homogenate binding assay.

### In vitro homogenate binding assay

100 µL of homogenized tissue (endocrine and exocrine pancreatic cell fractions from human and mouse as well as mouse brain cortex and spleen) were incubated for 30 min at RT with 2.5 MBq/mL [^11^C]FMZ (50 nM) in a final suspension with PBS (pH 7,4) for a total volume of 200µL in a polystyrene round bottom tube. At the end of the incubation time, the samples were filtered via a Brandel harvester for Liquid Scintillation Counter using cold PBS through a Whatman GF/C filter of 1.2 µm particle retention (# 1822021; Cytivia). Filter patches containing cells were dried for 30 min. Radioactivity was measured in a well counter (Uppsala Imanet AB, Uppsala, Sweden) and corrected for radioactive decay. Sample measurements were performed either in duplicate or in triplicate and normalized according to protein concentration**.**

### In vitro autoradiography

Fresh frozen cell fractions (isolated islets, isolated exocrine pancreas) and tissue (pancreas, spleen and cortex) from mouse, pig or human non-diabetic and T2D donors, were embedded into O.C.T (Q Path mounting media, VWR) and sectioned on cryotome (Cryostat NX70, ThermoFisher) in 10 µm or 20 µm thickness. Slices were mounted on SuperFrost Plus glass slides (ThermoFisher) and kept at -80 °C prior to the experiment. Duplicates of tissue and cell fraction sections were immersed in 150 mL of 50 mM cold PBS (Ph7.4) for 10 min. The cell and tissue slides were then incubated for 30 min in cold PBS (pH 7.4) containing low (2,5 nM) and high (5 nM) concentrations of [^11^C]FMZ corresponding to radioligand activities of 50 MBq and 90 MBq respectively. Three washing steps of 2 min in cold PBS and 1 min in distilled water were performed before drying the cell and tissue slides at 37 °C for 10 min and exposing them for 60 min on a BAS-IP storage phosphor screen (Fujifilm). Reference samples of known radioactivity were included simultaneously with the sections. The resulting digital image readout was obtained using an Amersham Typhoon storage phosphor imager (GE healthcare). Aliquots of each cold PBS (pH 7.4) bath containing low or high concentrations of [^11^C]FMZ were taken in triplicate to measure [^11^C]FMZ activity remaining at the end of the experiment and used for data analyses.

### Immunohistochemistry

Sections of human, pig, mouse fresh frozen cell fractions (isolated islets and exocrine pancreas) and tissue (pancreas, spleen and cortex) used for Autoradiography assay (with high dose of [^11^C]FMZ) were taken for a hematoxylin and eosin staining. The cell and tissue sections stored in − 20 °C after ARG assay were placed at room temperature for 15 min, incubated after rehydration in hematoxylin for 5 min, immersed in running water for 10 min, rinsed in distilled water for 10 s, dipped in eosin for 45 s and briefly rinsed in distilled water before dehydration and mounting.

Freshly isolated islets from cadaveric donors (Suppl. Table S1) were harvested, washed in PBS 0.5% BSA, fixed in 4% paraformaldehyde for 1 h at 4 °C, cryoprotected in 15% sucrose solution in PBS for 1 h, then in 30% sucrose solution in PBS overnight. Human islets were then embedded in OCT on dry ice and sectioned in 8 µm. Pig and mouse organs (pancreas or cortex) were harvested, fixed with 10% formalin overnight, washed in water for Three hours, transferred into 70% ethanol solution for 48 h and embedded in paraffin before sectioning in 8 µm. Paraffin-embedded human, pig and mouse tissue sections or OCT-mounted human or mice isolated islets were incubated 30 min or 1 h respectively with blocking buffer (10% fetal bovine serum diluted in Tris–HCl pH7.4, 0.15 M NaCl, 0.1%Triton-X100 [TBST]). For mouse monoclonal primary antibodies, sections were additionally blocked with mouse in mouse reagent (60 min, diluted in PBS) (Vector labs #MKB-2202). Cell fraction or tissue sections were incubated with primary antibodies (Suppl. Table S2) overnight at + 4 °C followed by washes and fluorochrome labelled secondary antibodies for 1 h at room temperature on paraffin-embedded sections or overnight at + 4 °C on OCT-embedded-sections (Suppl. Table S2).

### Image analysis

PET imaging data were analyzed with manual segmentation of the brain, pancreas and spleen on sequential transaxial projections using PBAS modeling tool (PMOD technologies LLC, Zurich, Switzerland). Data analyses for PET / CT imaging and quantifications were performed as previously described (Cheung et al., 2021). For Hematoxylin & Eosin staining performed on human, mouse and pig tissue following the autoradiography assay, images were acquired by scanning the complete slide to obtain a total overview of the tissue at × 4 magnification, using a digital pathology slide scanner (PathScan Enabler IV; Meyer Instrument). For higher magnification, H&E staining of the biological tissue was viewed with a Leica microscope LMD6000 (Leica Microsystems) and image acquisition was performed using LMD software.

### Statistical analyses

Unless otherwise indicated, statistical analyses were performed and graphics were produced using GraphPad Prism version 9 software (GraphPad Software, Boston, MA, USA). Non-parametric analysis was used to calculate statistical significance, which was defined as p-values < 0.05. All values are presented as mean ± SEM.

## Supplementary Information

Additional file 1.

## Data Availability

The datasets used and/or analysed during the current study are available from the corresponding author on reasonable request.

## Abbreviations

CNS: Central nervous system
[^11^C]FMZ: [^11^C]flumazenil
GABA: γ-Aminobutyric acid
GABA_A_Rs: γ-Aminobutyric acid A receptors
H&E: Hematoxylin and Eosin
PET: Positron emission tomography
T1D: Type 1 diabetes
T2D: Type 2 diabetes
