# Supplementary material for: PET imaging of GABAA receptors in pancreatic islets by [11C]flumazenil
Source: EJNMMI Res. 2024 Dec 2;14:122. doi: 10.1186/s13550-024-01176-5 (PMC11612099; doi:10.1186/s13550-024-01176-5)
Supplement: Supplementary file 1 — Additional file 1. [file 13550_2024_1176_MOESM1_ESM.docx]

**SUPPLEMENTARY DATA**

**PET imaging of GABA_A_ receptors in pancreatic islets by [^11^C]flumazenil**

**Supplementary Figure Legends**


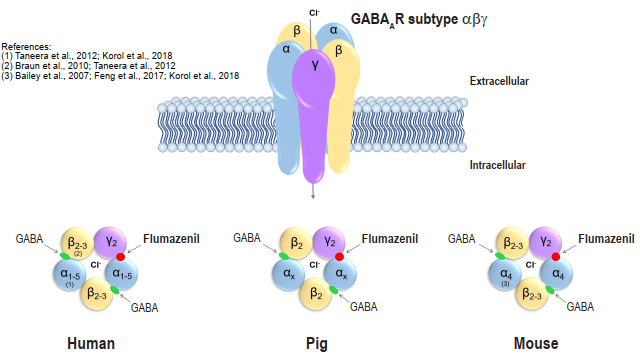
**Suppl. Figure S1:** The structure of GABA_A_ receptor subtypes in human, pig and mouse endocrine pancreas, and the location of the binding site of relevant ligands including flumazenil.


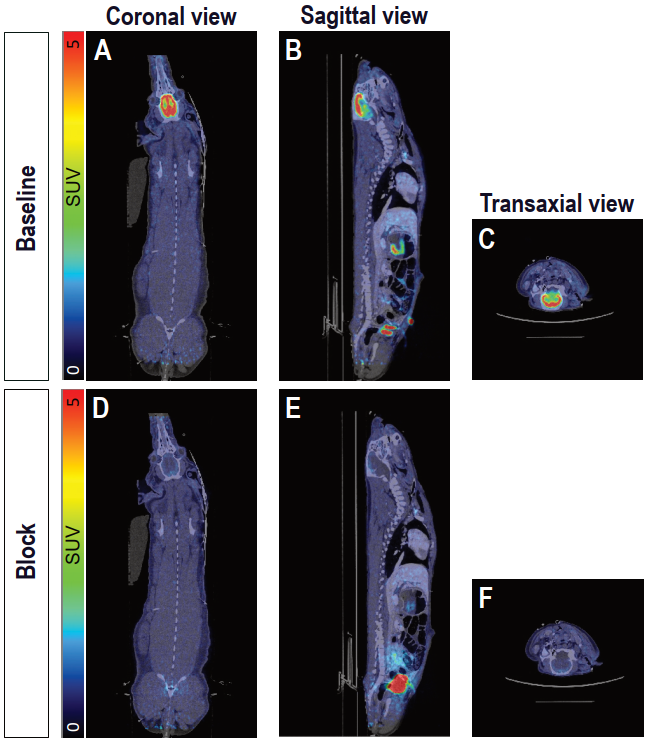


**Suppl. Figure S2: Whole body PET/CT imaging of GABA_A_Rs availability in pig using [^11^C]FMZ.** Coronal (**A,D**), sagittal (**B,E**) and transaxial (**C,F**) views of fused PET/CT scans from [^11^C]FMZ-injected pig (SUV=5) (**A-F**) showing the averaged signal from 60-90 min after [^11^C]FMZ injection, in the whole body at baseline (**A-C**) and after blocking with cold flumazenil (**D-F**).


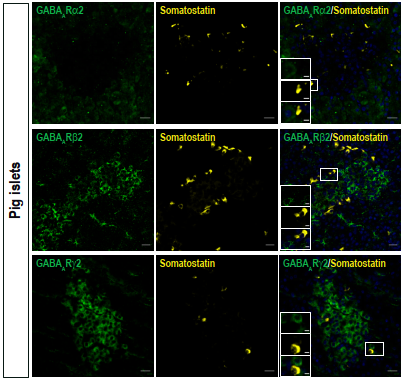


**Suppl. Figure S3: Expression profile of GABA_A_R α2, β2, γ2 subunits in pancreatic islet δ cells of control pigs.** Pancreatic tissue from control pigs (n=4) was co-stained with antibodies for GABA_A_R subunits α2 (top panel), β2 (middle panel), γ2 (bottom panel) (green), somatostatin (yellow), nuclei (blue). Scale bar 20 µm and 5 µm for magnification.


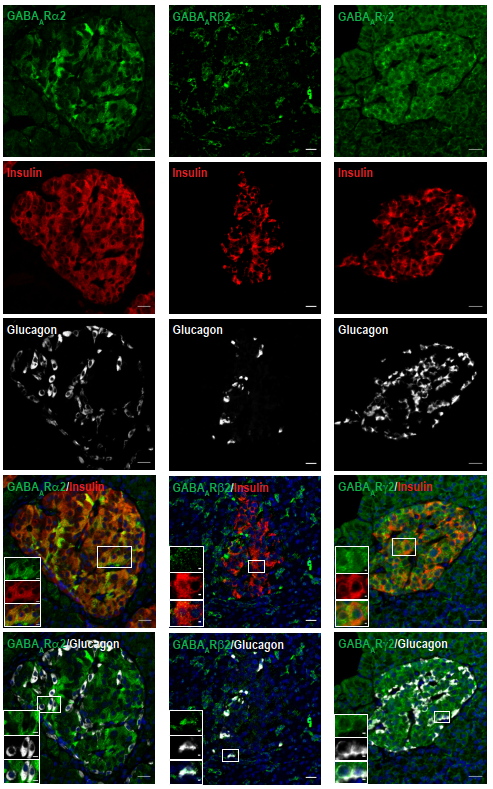


**Suppl. Figure S4: Expression profile of GABA_A_R α2, β2, γ2 subunits in ND human pancreatic islet β and α cells**. Human pancreatic tissue from non-diabetic organ donors (n= 3) was co-stained with antibodies staining GABA_A_R subunits α2 (left panel), β2 (middle panel), γ2 (right panel) (green), insulin (red), glucagon (white), nuclei (blue). Scale bar 20 µm and 5 µm for magnification.


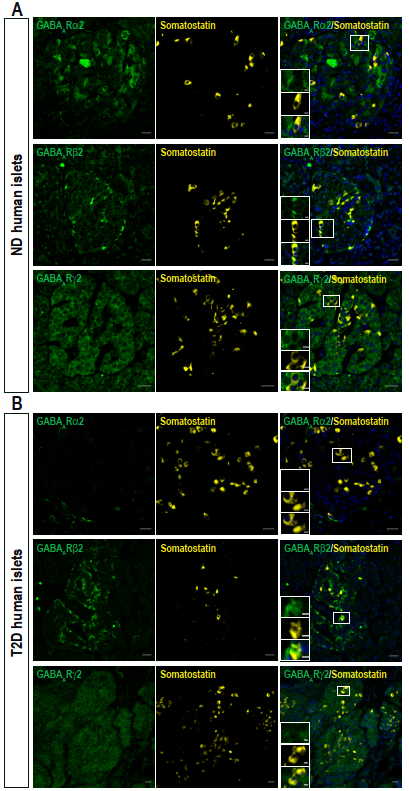


**Suppl. Figure S5: Expression profile of GABA_A_R α2, β2, γ2 subunits in ND and T2D human pancreatic islet δ cells.** Human pancreatic tissue sections from healthy donors (n= 3) (**A**) and two different T2D donors (n=1 for α2 and γ2 and n=1 for β2).) (**B**), were co-stained with antibodies staining GABA_A_R subunits α2 (top panel), β2 (middle panel), γ2 (bottom panel) (green), somatostatin (yellow), nuclei (blue) . Scale bar 20 µm and 5 µm for magnification.


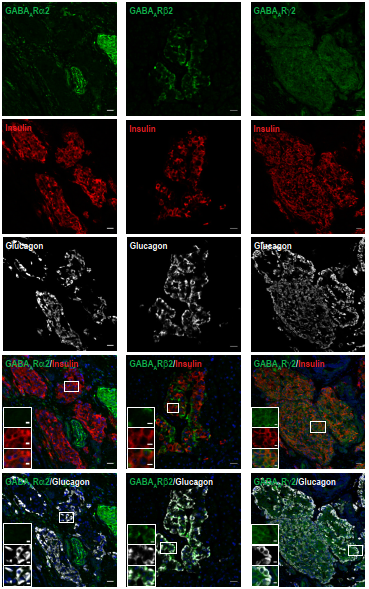


**Suppl. Figure S6: Expression profile of GABA_A_R α2, β2, γ2 subunits in T2D human pancreatic islet β and α cells**. Human pancreatic tissue from T2D donors was co-stained with antibodies staining GABA_A_R subunits α2 (left panel), β2 (middle panel), γ2 (right panel) (green), insulin (red), glucagon (white), nuclei (blue). Pancreatic sections from two different T2D donors were used for the immunohistochemistry (n=1 for α2 and γ2 and n=1 for β2). Scale bar 20 µm and 5 µm for magnification.


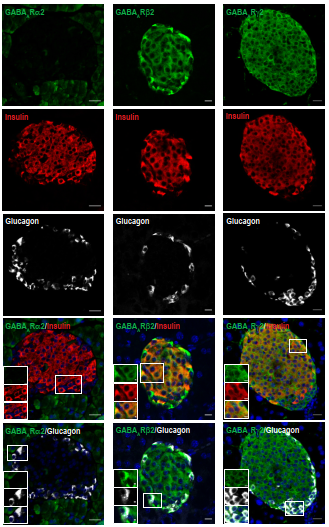


**Suppl. Figure S7: Expression profile of GABA_A_R α2, β2, γ2 subunits in mouse pancreatic islet β and α cells**. Mouse pancreatic tissue (n=6) was co-stained with antibodies staining GABA_A_R subunits α2 (left panel), β2 (middle panel), γ2 (right panel) (green), insulin (red), glucagon (white), nuclei (blue). Scale bar 20 µm and 5 µm for magnification.


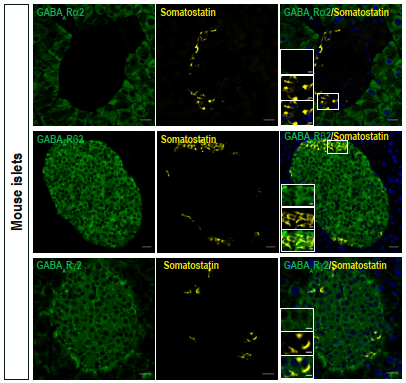


**Suppl. Figure S8: Expression profile of GABA_A_R α2, β2, γ2 subunits in mouse pancreatic islet δ cells.** Mouse pancreatic tissue sections (n= 6) were co-stained with antibodies staining GABA_A_R subunits α2 (top panel), β2 (middle panel), γ2 (bottom panel) (green), somatostatin (yellow), nuclei (blue). Scale bar 20 µm and 5 µm for magnification.

**Supplementary Tables**

**Supplementary Table S1**. **Donor pancreatic tissue and isolated islets characteristics**

| **Donor #** | **Specimen** | **Age**  **(y)** | **Gender** | **BMI**  **(kg/m^2^)** | **Diabetes state** | **HbA1c**  **(mmol/mol)** |
| --- | --- | --- | --- | --- | --- | --- |
| 1 | pancreas | 59 | male | 26.1 | Non-diabetic | _ |
| 2 | pancreas | 41 | female | 22.9 | Non-diabetic | 38.0 |
| 3 | pancreas | 52 | female | 33.0 | T2D | 65.0 |
| 4 | pancreas | 78 | female | 22.9 | T2D | 65.0 |
| 5 | islets + exocrine tissue | 61 | female | 24.7 | Non-diabetic | 25.0 |
| 6 | pancreas | 61 | female | 24.7 | Non-diabetic | 25.0 |
| 7 | pancreas | 52 | male | 26.9 | Non-diabetic | 37.0 |
| 8 | pancreas | 54 | male | 35.1 | T2D | 47.0 |
| 9 | islets + exocrine tissue | 49 | male | 23.6 | Non-diabetic | 33.0 |
| 10 | islets + exocrine tissue | 58 | male | 32.8 | Non-diabetic | _ |
| 11 | islets + exocrine tissue | 37 | female | 44.4 | Non-diabetic | 42.0 |
| 12 | islets + exocrine tissue | 67 | female | 29.0 | Non-diabetic | 33.0 |
| 13 | islets + exocrine tissue | 53 | female | 18 | Non-diabetic | 36.0 |

**Supplementary Table S2**. **Primary and secondary antibodies used for immunohistochemistry**

| **Antigen** | **Species** | **Supplier** | **Dilution** |
| --- | --- | --- | --- |
| GABA_A_Rα2 | Rabbit | Synaptic Systems, # 224 103 | 1:200 |
| GABA_A_Rβ2 | Rabbit | Abcam, # ab186875 | 1:100 |
| GABA_A_Rγ2 | Rabbit | Synaptic Systems, # 224 003 | 1:100 |
| Glucagon | Mouse | Sigma-Aldrich; # G2654 | 1:300 |
| Insulin | Guinea pig | Fitzgerald, Acton, MA, USA; # 20-IP30 | 1:500 |
| Somatostatin | Rat | Bio-Rad; # 8330 | 1:200 |
| Alexa Fluor® 594 AffiniPure Donkey Anti-Guinea Pig IgG (H+L) |  | Jackson ImmunoResearch; 706-585-148 | 1:300 |
| Alexa Fluor® 488 AffiniPure Donkey Anti-Rabbit IgG (H+L) |  | Jackson ImmunoResearch; 711-545-152 | 1:300 |
| Alexa Fluor® 647 AffiniPure Donkey Anti-Mouse IgG (H+L) |  | Jackson ImmunoResearch; 715-605-150 | 1:300 |
| Alexa Fluor® 647 AffiniPure Donkey Anti-Rat IgG (H+L) |  | Jackson ImmunoResearch; 712-607-003 | 1:300 |

**Supplementary Table S3**. **Distribution of GABA_A_R α2, β2, γ2** **subunits in endocrine pancreas from different species**

|  | **Glucagon-producing α cell** | **Insulin-producing β cell** | **Somatostatin-producing δ cell** |
| --- | --- | --- | --- |
| **Pig** | **β2, γ2** | **β2, γ2** | **/** |
| **ND donors** | **β2, γ2** | **α2, β2, γ2** | **β2, γ2** |
| **T2D donors** | **β2, γ2** | **γ2** | **β2** |
| **Mouse** | **β2, γ2** | **β2, γ2** | **β2, γ2** |
